# Early insights from the routine use of patient reported outcome measures in elective hip and knee arthroplasty at a public teaching hospital in South Australia

**DOI:** 10.1186/s41687-024-00807-8

**Published:** 2024-11-12

**Authors:** Samuel P. Goldsmith, Paul N. Karayiannis, Louisa M. Edwards, Barbara Toson, Freeda D’Mello, Emma Jackman, Christopher John Wilson, Anthony Samson

**Affiliations:** 1 College of Medicine and Public Health, Flinders University, Level 5, Room 5E209, Bedford Park, Adelaide, 5042 South Australia; 2 Department of Orthopaedic and Trauma Surgery, Flinders Medical Centre, Adelaide, South Australia

**Keywords:** Patient-reported outcome measures, PROMs, Hip-arthroplasty, Knee-arthroplasty, Oxford-knee score, Oxford-hip score, Forgotten joint score, EuroQol-Visual Analogue Scale

## Abstract

**Introduction:**

For advanced osteoarthritis of the knee and hip, the most clinically effective treatment remains total-knee arthroplasty (TKA) and total-hip arthroplasty (THA). Success of these surgeries have traditionally been appraised by economic and volume-based measures. There has been a shift towards the use of patient reported outcome measures (PROMs) to quantify success and guide treatment. The present study provides analysis of three PROMS which have been validated for use in orthopaedic settings; the Oxford Knee Score (OKS), Oxford Hip Score (OHS), Forgotten Joint Score (FJS), and the EuroQol-Visual Analogue Scale (EQ-VAS) - a non-disease specific measure of health. PROMs were completed pre-operatively, 6-weeks, and 1-year after elective TKA and THA undertaken in 2018 in a public teaching hospital in South Australia. Post-operative satisfaction/dissatisfaction was measured using a 5-point Likert scale and was collected at the same 6-week and 1-year points.

**Results:**

PROMs were collected from 285 eligible elective knee-arthroplasty, and 205 elective hip-arthroplasty patients. There was significant average improvement, greater than minimal clinical important differences between pre-operative and 1-year post-operative scores for all three PROMs tools. Inter-PROM correlation was strongest between FJS and OKS at 1-year post TKA (r_s_ = 0.722), and between FJS and OHS in post-THA at the same interval (r_s_ = 0.609). TKA patients with higher pre-surgical 10-year mortality were weakly associated with lower pre-operative OKS score (r_s_ = 0.169). BMI was weakly negatively associated with pre-operative and 6-week post-operative EQ-VAS scores (r_s_ = -0.291 and r_s_ = -0.149 respectively). Post-TKA satisfaction was 77.2% at 1-year, and THA 88.5% at the same interval.

**Conclusion:**

This study provides an early insight from the use of the OKS, OHS, the EQ-VAS and the FJS as PROMs in primary TKA and THA at our centre. All PROMs demonstrate significant increase (improvement) at both 6-week and 1-year post-operative intervals, relative to pre-operative scores. The FJS demonstrated good sensitivity. Pre-existing co-morbidities do not appear to have any significant relation with post-operative PROMs collected in this study.

**Supplementary Information:**

The online version contains supplementary material available at 10.1186/s41687-024-00807-8.

## Introduction

Symptomatic osteoarthritis (OA) affects one in five adults older than 45 years and one third of adults older than 75 years, often resulting in severe pain, loss of function and reduced workplace productivity [1]. Joint replacement remains the most clinically effective treatment for end stage disease, resulting in an increased demand for total knee arthroplasty (TKA) and total hip arthroplasty (THA) by 27% and 33% respectively between 2009 and 2018 [1].

Outcomes of hip and knee replacement surgeries have traditionally been measured by economic and volume-based healthcare delivery [2] – often in the form of cost-effectiveness analysis. These measures weigh the cost-effectiveness of providing joint replacement against costs associated with other management, between types of prosthesis or surgical approach. Systematic reviews have demonstrated despite the high costs associated with THA and TKA, they remain highly cost-effective treatments for OA [3–4]. However recent adoption of patient-reported outcome measures (PROMs) reflects a shift towards person-centred measures of healthcare across the industry. Broadly, PROM questionnaires may be used for describing a person’s quality of life (QoL), quantifying pain status, impact on activities of daily living and general perspective on illness burden [5].

The International Consortium for Health Outcome Measurement (ICHOM) documents a standard set of important outcomes and measurements for hip and knee osteoarthritis. These include disease control (need for surgery, treatment progression, re-operation, or revision), acute complications (mortality and readmissions) and patient-reported health status (pain, physical function, work status, health-related QoL and overall satisfaction) [6]. Several PROMs have been validated for use in this cohort by both ICHOM and other research bodies. The Oxford Knee Score (OKS) and Oxford Hip Score (OHS) are widely implemented PROMs tools for the assessment of pain and function in pre- and post-operative knee and hip arthroplasty patients with osteoarthritis. A 2016 review identified the OKS and OHS as the psychometric measures with the most complete evidence supporting their use in patients undergoing hip or knee replacement surgeries, when compared to 32 other PROMs [7]. The OKS and OHS have also been validated for use in the Australian Orthopaedic context [8].

Another PROM, the Forgotten Joint Score (FJS) was developed with intention of introducing a patient’s ability to forget their artificial joint in everyday life [9]. A 2020 systematic review of 13 articles showed strong evidence of good test-retest reliability regarding the FJS and moderate evidence of internal consistency [10]. Consensus-based standards for the selection of health Measurement Instruments (COSMIN) checklist analysis during the tool’s development found content validity was ‘good’ [10], although no other articles have repeated this analysis. However, the Forgotten Joint Score has not been validated for use in Australian populations [8]. The EQ-5D-5 L tool is recommended by the ICHOM and is also validated for use in Australia for hip and knee osteoarthritis patients. It does not specifically focus on their joint [6] but is rather as a measure of health utility. In part one, patients are instructed to rank on a scale of 1 to 5 their mobility, self-care, usual activities, pain/discomfort, anxiety/depression. Part two of the EQ-5D-5 L is the EuroQol-Visual Analogue Scale (EQ-VAS) measure, which asks patients to rank their health on a 20 cm, vertical scale from the ‘best health imaginable’ to ‘worst health imaginable’. The EQ-VAS has been reported to have a predictable and consistent relationship with the EQ-5D profile and other PROMs systems in the NHS [11] whilst having a lower questionnaire burden. This is particularly important for patients already completing other PROMs – such as in the present study.

Satisfaction and dissatisfaction are commonly measured on either a 10 or 5-point Likert scale, wherein patients self-report feeling one of; ‘very satisfied (5)’, ‘satisfied (4)’, ‘neither satisfied nor unsatisfied (3), ‘unsatisfied (2)’ or ‘very unsatisfied (1)’ [12]. Previous studies have reported high rates of post-operative dissatisfaction in TKA patients, with rates as high as 20% [13]. There is a growing push in Australia for nationwide collection of PROMs to shed further light on these issues [14]. Reduced post-operative joint pain, better functional outcomes and appropriate pain relief have been linked to increased satisfaction [15].

Modification of post-operative protocols, in conjunction with PROM/satisfaction could identify factors that increase patient satisfaction/decrease dissatisfaction [16]. Post-operative protocols utilized in the treatment of patients from this current dataset are day 0 mobilization, regular analgesia as well as a pre- and post-operative physiotherapy mobility assessment and guidance (See Appendix 1). This study will analyse the 6-week and 1-year outcomes following elective hip and knee arthroplasty, using validated PROM scores and a satisfaction scale, performed by our department. This allows us to understand our own performance and facilitates comparison with other centres. Our secondary aim is to show whether modifiable patient factors or standardized surgical and post-operative pain management protocols have correlation with, and pre- and post-operative PROM scores and dissatisfaction levels. These scores allow us to create a point of comparison for future study with national data, from the Australian Orthopedic Association National Joint Replacement Registry (AOANJRR).

## Methods

Ethics approval was provided by application to the Southern Adelaide Local Health Network (SALHN) Office for Research (reference number 329.17). All patients above the age of 18 years who underwent elective, planned primary knee and hip arthroplasty in 2018 were invited to participate in the study at their immediate pre-operative assessment appointment (two weeks pre-operatively). Patients were excluded if they were unable to reliably communicate in the English language or unable to consent due to impairment in cognitive functioning or decision-making capacity. Patient information was deidentified prior to data analysis. Data from elected PROMs tools in consenting patients were thus collected pre-operatively and at 6-weeks, and 12-months post-operatively during routine appointments or mailed questionnaires. Peri-operative antibiotics were administered per SA Health guidelines for Orthopaedic and Spinal Surgery (see Appendix 2) to reduce the risk of post-operative infection. All post-operative clinical protocols–post-operative physio assessment and analgesia were adhered to (see Appendix 1).

PROMs data collected from all TKA patients included the Oxford Knee Score, the Forgotten Joint Score and the EuroQoL-5D-5 L(including EQ-VAS). The Oxford Hip Score, as well as the FJS, EQ-5D-5 L (including EQ-VAS) were collected instead from THA patients. The EQ-VAS was scored on a scale of 0-100, where 100 indicates that on the day of reporting, the patient had overall, the best health imaginable, and 0 indicated the worst health available. The OKS and OHS are scored out of 48, where a score of 48 indicates a patient was least symptomatic, and 0 that the patient was the most symptomatic possible on the scale [17–18]. A raw score for the FJS is calculated by adding the scores from each question. The raw score ranges from 12 to 60, where 60 represents the patient is most aware of joint pain, and 12 they are the least aware. This was then transformed into a 0-100 scale, and reversed, such that a score of 100 represents a patient who is least aware of their joint (i.e. higher is better).

At the same time as completing PROMs, patients scored satisfaction with their surgery on a 5-point Likert scale. On this scale a score of; 1: indicated the patient was very dissatisfied, 2: that the patient was dissatisfied, 3: indicated neither satisfaction nor dissatisfaction, 4: that the patient was satisfied, and 5 that the patient was very satisfied with their surgery. Likelihood to have another operation and likelihood to recommend surgery to another person was also recorded using the same five-point scale. Prior to surgery each patient basic demographic data collected; gender, age, weight, and body mass index (BMI). Charleston Comorbidity Index (CCI) was also calculated following methods published with the index [19]. A patients CCI is used to calculate an estimated 10-year survival percentage (i.e. higher number indicates better survival) [19]. 10-year survival was used for all analysis involving CCI measures.

All arthroplasties were undertaken with consultant supervision through either a posterior or anterior approach for hip replacements, more commonly with a hybrid construct using one of the 5 most common implants in the Australian registry. Knee arthroplasties similarly were undertaken predominantly with cemented cruciate retaining constructs. Complicated primaries requiring augments/hinges/dual mobility cups or constraint were not excluded from analysis.

### Statistical analyses

PROMs were scored according to their published scoring manuals and guidelines [20–23]. CCI, and CCI mortality was calculated according to published manuals and guidelines [19].

Data was assessed for normality using Shapiro-Wilk’s test, and correlation calculated with Spearman’s Rho where normality was violated. Correlation strength was interpreted as poor if *r* < 0.3, fair where 0.3 < *r* < 0.5, moderately strong where 0.6 < *r* < 0.8, and very strong when *r* > 0.8 [24]. Mixed linear models with random intercept for each patient was used to analyse the trend over time of each PROM outcome (pre-operative, 6-weeks and 1-year post-operation). Covariates analysed in these models included BMI, weight, and CCI. Patients with incomplete datasets were removed for modelling listwise. A robust variance estimator adjustment was used in the case of non-normally distributed residuals.

## Results

**Fig. 1:**
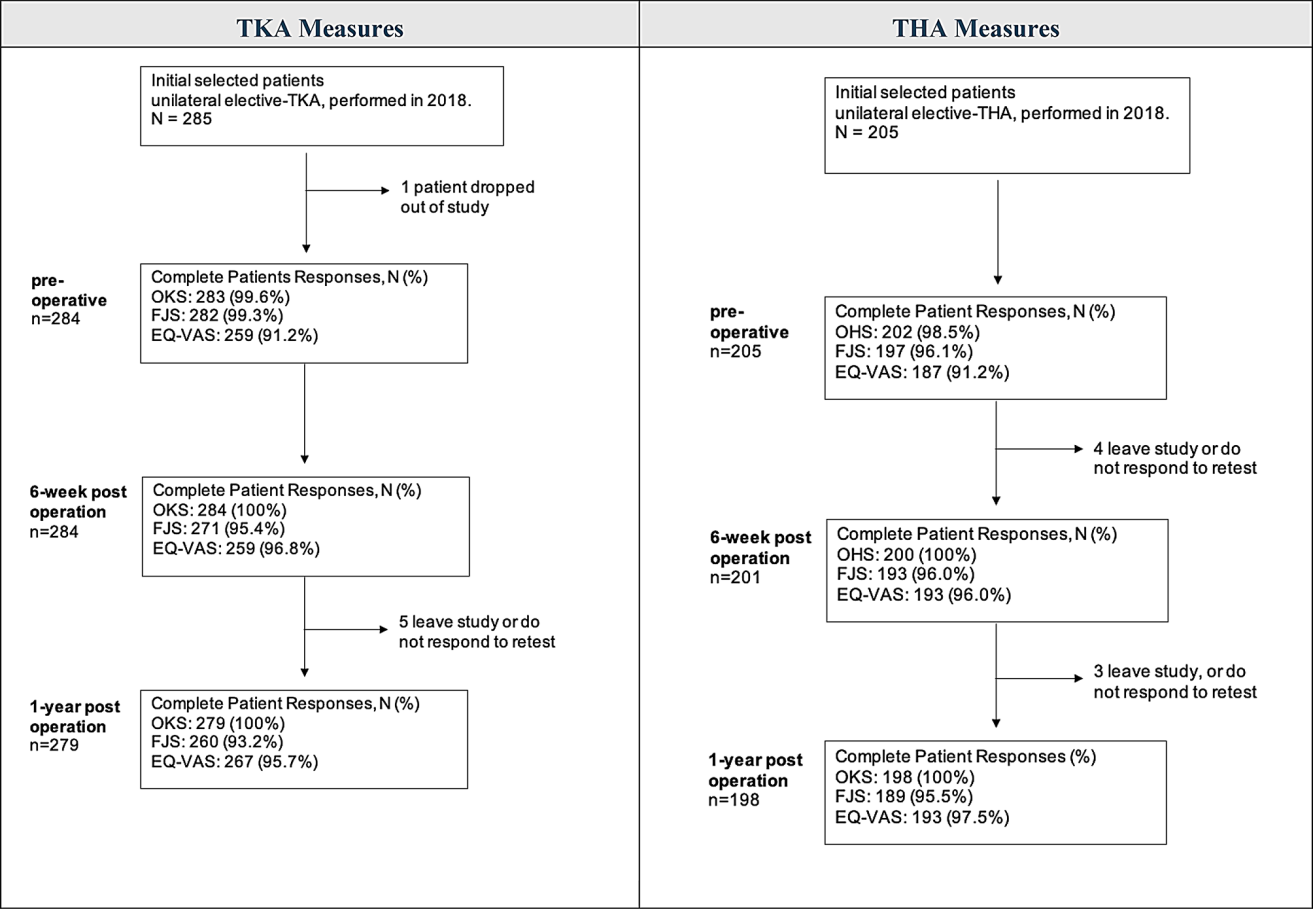
TKA and THA cohort participation, drop out and completion between initial consultation, pre-op consultation, 6-weeks post-operation, and 1-year post-operation

316 patients underwent elective primary-TKA and 247 elective primary-THA in 2018 at our centre. All were invited to participate in the study at their pre-operative assessment, however were excluded if unable to reliably communicate in the English language or had a level of cognitive impairment that would interfere with PROM collection. Data presented is from 285 knee arthroplasty patients and 205 hip arthroplasty patients who completed at least pre-operative PROMS. Within the TKA group; one patient had withdrawn from the study by the preoperative stage, and six by 1-year. In the THA group, five patients had withdrawn by 6-weeks post-operation, and seven by 1-year post-operation (Fig. 1). There were more females in both groups, with 167 in the knee group (58.6%) and 116 in the hips group (56.6%). Average age of the knee participants was 69.10 (SD 9.70) years and for the hips, 69.23 (SD: 11.23) years. Average BMI for the knee group was 32.03 kg/m2 (SD: 5.65) and 30.124 kg/m2 (5.86) for the hips; both classifying as “obese” according to Australian health standards [25]. See Table 1 for participant characteristics.

**Table 1:** TKA and THA patient cohort demographics descriptive statistics – age, weight, BMI, sex, complication rate

			TKA			THA		
			Age	Weight	BMI	Age	Weight	BMI
**Total N**			285	285	285	205	191	189
**Mean**			69.10	88.09	32.03	69.23	85.51	30.124
**Median**			70.00	89.00	32.00	70	81	29
**Std. Deviation**			9.70	18.78	5.65	11.23	22.77	5.86
**Range**			47.00	105.20	31.40	57	137	29
**Minimum**			44.00	33.90	19.60	39	46	18
**Maximum**			91.00	139.10	51.00	96	183	47.4
**Sex**	**F: N (%)**		167 (58.6%)			116 (56.6%)		
	**M: N (%)**		118 (41.4%)			89 (43.4%)		
**Complication**	**Y: N (%)**		230 (80.7%)			181 (88.3%)		
	**N: N (%)**		55 (19.3%)			24 (11.7%)		

Of the TKA patients who completed both pre-operative and 1-year post-operative OKS PROMs, 93.5% (*n* = 261) reported some degree of improvement, 4.3% (*n* = 12) worsened, and 2.15% (*n* = 6) of patients had the same score (Table 2). Mean difference in OKS was 9.779 at 6-weeks (*p* < 0.001), and 16.864 (*p* < 0.001) at 1-year post-operation.

**Table 2:** TKA cohort OKS, FJS and EQ-VAS PROM score descriptive statistics at pre-operative, 6-week post-operative, and 1-year post-operative intervals

PROM and Interval		*N* valid (missing)	Mean Score (+/- S.D)	Lower Quartile	Median	Upper Quartile	Range
**Oxford Knee Score**	Pre-operation	283 (2)	17.45 (± 7.26)	13	17	23	39
	6-weeks post	284 (1)	26.85 (± 8.96)	22	27	33	43
	1-year post	279 (6)	34.12 (± 9.87)	28	35	42	17
**Forgotten Joint Score**	Pre-operation	282 (3)	13.96 (± 16.51)	2.08	10.42	20.83	97.92
	6-weeks post	268 (17)	31.90 (± 24.58)	10.42	29.17	50	93.75
	1-year post	271 (14)	49.60 (± 29.7)	27.08	50	76.56	100
**EQ-VAS Score**	Pre-operation	259 (26)	68.37 (± 19.40)	60	70	85	95
	6-weeks post	273 (12)	73.24 (± 17.67)	65	80	90	80
	1-year post	267 (18)	78.80 (± 15.86)	70	80	90	80
**Change at 1-year**	**N of change in PROM score at 1-year post-op**						
	**Measure**	Improved, n (%)	Worsened, n (%)	Same, n (%)		Total n of patients	
	OKS	261 (93.5)	12 (4.3)	6 (2.15)		279	
	FJS	214 (79.0)	19 (7.0)	11 (4.0)		271	
	EQ-VAS	178 (72.1)	47 (19.0)	22 (8.9)		247	

**Fig. 2:**
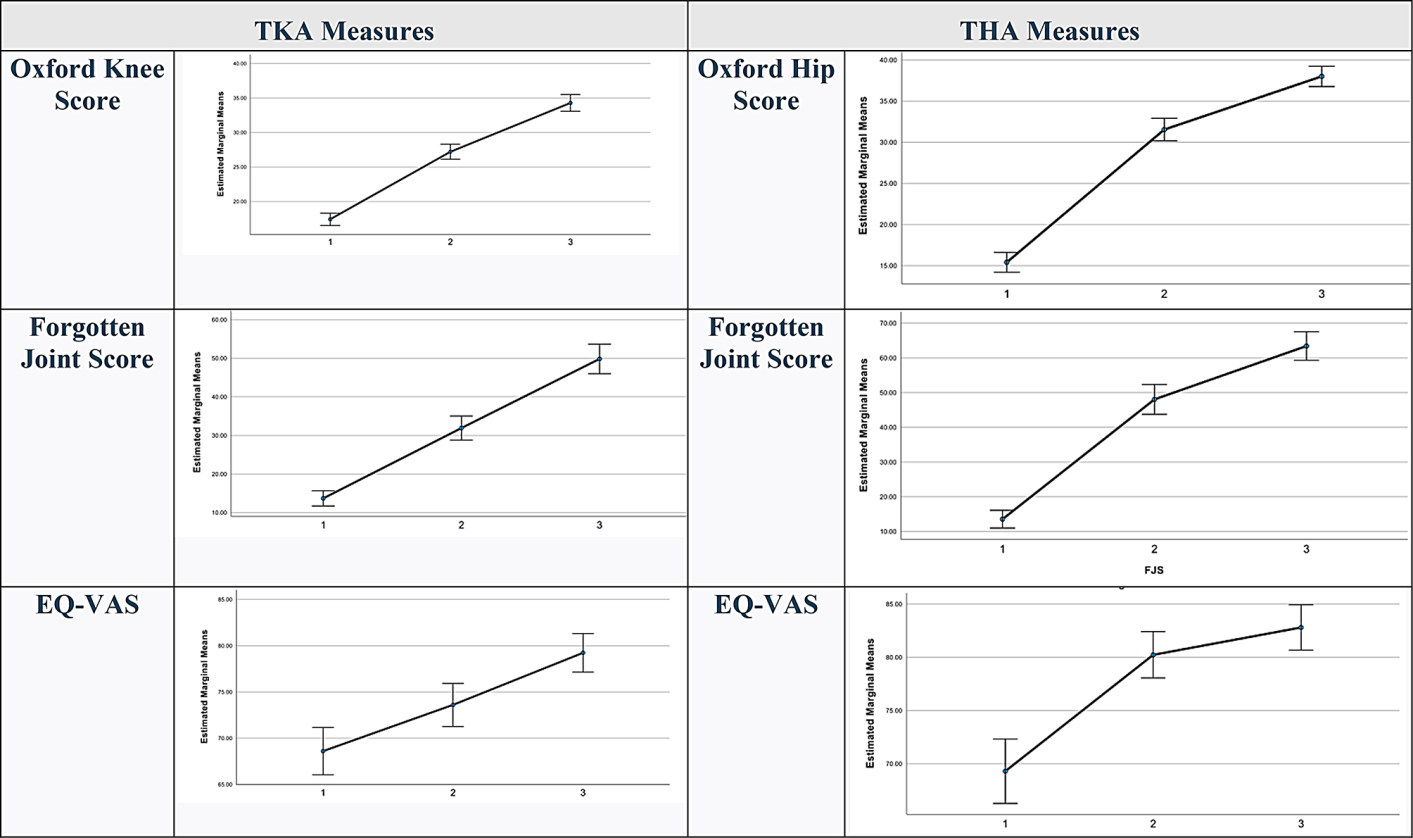
Marginal Means (+ 95% CI) of Oxford Knee/Hip Score, Forgotten Joint Score and EQ-VAS at (1) Pre-operative (2) 6-week post-operative and (3) 1-year post-operative Intervals

In the FJS dataset, at 1-year post-operation, 79% (*n* = 214) of patients had improved scores, and 19% of patients worsened. Mean difference in FJS was 34.504 and 49.871 at 6-weeks and 1-year post-THA intervals respectively (*p* < 0.001). 72.1% (*n* = 178) of TKA patients reported some improvement in overall health 1-year post operation on the EQ-VAS. Mean difference in this measure was 5.005 (*p* = 0.003) at 6-weeks, and 10.635 (*p* < 0.001) at 1-year (appendix 3). At 1-year post-THA, 97.5% (*n* = 192) of patients scored higher on the OHS, and a mean difference relative to pre-operative scores of 22.594 (*p* < 0.001) was found. 94.2% (*n* = 178) of patients demonstrated improvement by 1-year with the FJS (Table 3). Mean difference in this measure and interval was 49.87 (*p* < 0.001) (Fig. 2). 82.8% (*n* = 149) of patients reported higher EQ-VAS 1-year post-THA relative to pre-operative scores. The mean difference in pre-operative and 1-year post-THA EQ-VAS was 13.5 (*p* < 0.001) (appendix 3).

**Table 3:** THA cohort OHS, FJS and EQ-VAS PROM scores descriptive statistics at; pre-operative, 6-week post-operative, and 1-year post-operative intervals

PROM and Interval		*N* valid (missing)	Mean Score (+/- S.D)	Lower Quartile	Median	Upper Quartile	Range
**Oxford Hip Score**	Pre-operation	202 (3)	15.42 (± 8.43)	9	16	21	40
	6-weeks post	200 (5)	31.32 (± 9.39).	25	32	38	47
	1-year post	198 (7)	38.09 (± 8.47)	34	40	44	48
**Forgotten Joint Score**	Pre-operation	197 (8)	13.84 (± 17.59)	2.08	8.33	19.79	93.75
	6-weeks post	193 (12)	48.09 (± 27.73)	25	43.75	70.83	100
	1-year post	189 (16)	63.25 (± 26.24)	47.92	66.67	83.33	100
**EQ-VAS Score**	Pre-operation	187 (18)	70 (± 20.56)	55	70	83.75	90
	6-weeks post	193 (12)	80 (± 14.62)	70	80	90	80
	1-year post	193 (12)	85 (± 13.86)	75	85	90	70
**Change at 1-year**	**N of change in PROM score at 1-year post-op**						
	**Measure**	**Improved**, **n (%)**	**Worsened**, **n (%)**	**Same**, **n (%)**		**Total n of patients**	
	OHS	192 (97.5)	5 (2.5)	0		197	
	FJS	178 (94.2)	10 (5.3)	1 (0.5)		189	
	EQ-VAS	149 (82.8)	25 (13.9)	16 (8.9)		180	

Weak correlation was identified between all pre-operative PROMs, in both knee and hip data sets. The strongest associations were between Oxford Knee Score and Forgotten Joint Score (r_s_ = 0.496) in TKA patients, and between Oxford Hip and Forgotten Joint Score in THA patients (r_s_= 0.459). Correlation between the EQ-VAS and the other three measures was weaker pre-operatively (Table 4). Post-operatively, moderately strong associations exist between the OKS and FJS at 1-year post-TKA (r_s_ = 0.722), and the OHS and FJS at 1-year post-THA (r_s_ = 0.609). Correlation between EQ-VAS and other measures is weaker (Table 4).

**Table 4:** PROM score correlation at; pre-operative, 6-week post-operative, and 1-year post-operative intervals

Correlation between pre-operative scores										
Knee Measures		OKS	FJS	EQ-VAS	Hip Measures		OHS		FJS	EQ-VAS
**OKS**	Correlation Coefficient (n)	1 (283)	0.496^**^ (280)	0.271^**^ (258)	OHS	Correlation Coefficient (n)	1 (202)		0.459^**^ (197)	0.363^**^ (187)
**FJS**	Correlation Coefficient (n)	0.496^**^ (280)	1 (282)	0.195^**^ (257)	FJS	Correlation Coefficient (n)	0.459^**^ (197)		1 (197)	0.262^**^ (182)
**EQ-VAS**	Correlation Coefficient (n)	0.271^**^ (258)	0.195^**^ (257)	1 (259)	EQ-VAS	Correlation Coefficient (n)	0.363^**^ (187)		0.262^**^ (182)	1 (187)
**Correlation between scores at 6-weeks post-operation**										
**Knee Measures**		**OKS**	**FJS**	**EQ-VAS**	**Hip Measures**			**OHS**	**FJS**	**EQ-VAS**
**OKS**	Correlation Coefficient (n)	1 (284)	0.407^**^(267)	0.279^**^ (272)	OHS	Correlation Coefficient (n)		1 (200)	0.539^**^ (193)	0.483^**^ (193)
**FJS**	Correlation Coefficient (n)	0.407^**^ (267)	1 (268)	0.301^**^ (258)	FJS	Correlation Coefficient (n)		0.539^**^ (193)	1 (193) 193	0.288^**^ (187)
**EQ-VAS**	Correlation Coefficient (n)	0.279^**^ (272)	0.301^**^(258)	1 (273)	EQ-VAS	Correlation Coefficient (n)		0.483^**^ (193)	0.288^**^ (187)	1 (193)
**Correlation between scores at 1-year post-operation**										
**Knee Measures**		**OKS**	**FJS**	**EQ-VAS**	**Hip Measures**			**OHS**	**FJS**	**EQ-VAS**
**OKS**	Correlation Coefficient (n)	1 (279)	0.722^**^ (271)	0.505^**^ (267)	OHS	Correlation Coefficient (n)		1 (198)	0.609^**^ (189)	0.408^**^ (193)
**FJS**	Correlation Coefficient (n)	0.722^**^ (271)	1 (271)	0.417^**^ (262)	FJS	Correlation Coefficient (n)		0.609^**^ (189)	1 (189)	0.428^**^ (186)
**EQ-VAS**	Correlation Coefficient (n)	0.505^**^ (267)	0.417^**^ (262)	1 (267)	EQ-VAS	Correlation Coefficient (n)		0.408^**^ (193)	0.428^**^ (186)	1 (193)

Correlation made using Spearman’s Rank Correlation

**. Correlation is significant at the 0.01 level (2-tailed)

*. Correlation is significant at the 0.05 level (2-tailed)

To determine the proportion of variance in FJS that may be explained by differences in 1-year post-operative function, patients were divided into three groups based on their Oxford Knee/Hip score. Low functioning was defined as (< 27), moderate (27–41) or high function (> 41) [26]. Eta-squared was 0.435 and 0.308 in the knee and hip-arthroplasty groups respectively (Table 5). Comparison between moderate and high OKS and OHS produced Cohen’s D of -0.85, and − 0.84 respectively.

**Table 5:** FJS by functional level means and effect (Eta-Squared and Cohen’s D) 1-year Post TKA/THA

Functional Level	Mean FJS (+/- S.D)	*N*	Groupwise Comparison	Cohen’s D	Eta-squared
1. Low (OKS < 27)	25.95 (27.62)	66	**1–2**	-0.76	0.435
2. Moderate (OKS 27–41)	44.55 (20.76)	128	**1–3**	-1.50	
3. High (OKS > 41)	78.27 (19.98)	77	**2–3**	-0.86	
1. Low (OHS < 27)	30.30 (26.04)	22	**1–2**	-1.19	0.308
2. Moderate (OHS 27–41)	59.81 (23.37)	94	**1–3**	-2.10	
3. High (OHS > 41)	77.63 (17.52)	72	**2–3**	-0.84	

In the knee dataset, 10-year mortality was poorly predictive for pre-operative OKS score (r_s_ = 0.17, p = < 0.01), and pre-operative EQ-VAS scores (r_s_ = 0.125, p = < 0.05). There was no significant association between 10-year mortality and FJS scores in the TKA dataset. Within the THA cohort there was no significant correlation between CCI-calculated survival and pre- or post-operative FJS, OHS or EQ-VAS score.

BMI was poorly negatively associated with OHS and EQ-VAS scoring in the hips dataset - where a higher BMI indicated lower pre-operative OHS (r_s_ = -0.162) and preoperative EQ-VAS means (r_s_ = -0.291) (appendix 4). There was no strong association between BMI and post-THA PROMs. There was poor association between BMI and PROMs scorings in the knee datasets (r_s_ = -0.155), both at pre- and both post- operative points. CCI was poorly predictive for OKS in the pre-operative setting (r_s_ = 0.169), and EQ-VAS (r_s_ = 0.125) (appendix 4).

**Fig. 3:**
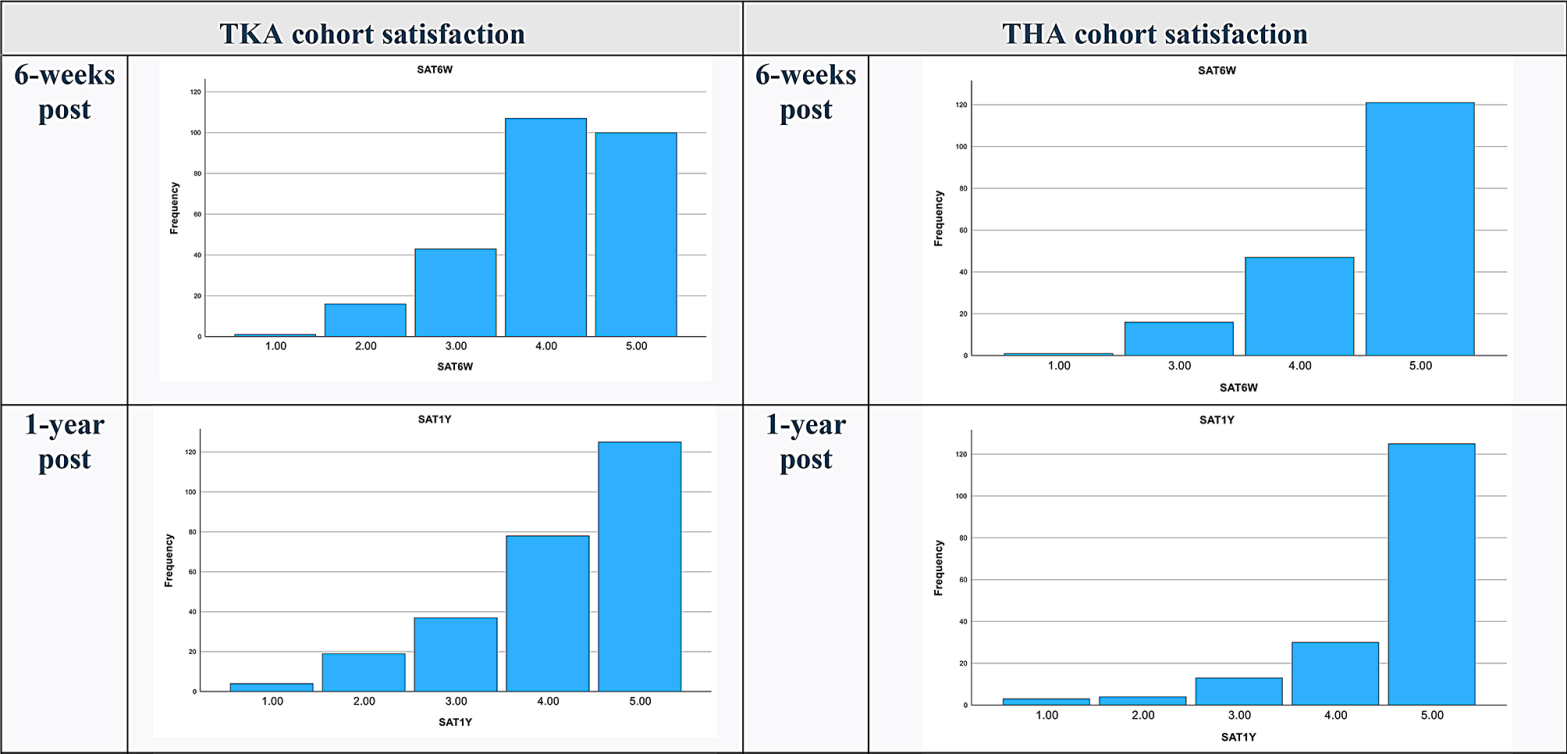
Post operative satisfaction-scale frequency, 6-weeks and 1-year post operation

The average satisfaction score 1-year post-TKA was 4.144 (from a scale of 1 to 5). the percentage of patients post-TKA reporting being either ‘satisfied’ or ‘very satisfied’ was 77.6% at 6-weeks and 77.2% at 1-year follow ups. THA cohort post-operative satisfaction at 6-week follow up was 90.8%, and 88.5% at 1-year (Fig. 3) Patient dissatisfaction (an aggregate of those who answered the question with either a 1 = ‘very unsatisfied or 2 = ‘unsatisfied’) post-TKA was 6.4% at 6-weeks and 8.7% at 1-year. Post-THA dissatisfaction was 0.5% at 6-weeks and 4.0% at 1-year. Complete response data for all categories is displayed in appendix 5.

Post-operative satisfaction was correlated with all PROM measures, at both post-operative capture points. In the THA cohort, the OHS demonstrated fair positive correlation with satisfaction at both 6-weeks and 1-year (*r* = 0.403 and *r* = 0.405, respectively) (Table 6). There was poor correlation between the FJS and satisfaction (*r* = 0.213 at 6-week, and *r* = 0.288 at 1-year), and EQ-VAS and satisfaction (*r* = 0.179 at 6-weeks and *r* = 0.364 at 1-year). Within the post-TKA group, generally correlation between satisfaction was highest at 1-year post-operation. All three PROM measures; OKS (*r* = 0.605), FJS (*r* = 0.523) and EQ-VAS (*r* = 0.413) were fair-moderately positively associated with satisfaction at this time (Table 6).

**Table 6:** Correlation between post-TKA and THA (at 6-weeks and1-year) PROM and satisfaction scores

		OHS	FJS	EQ-VAS	OKS	FJS	EQ-VAS
**Post-operative satisfaction**	**6-weeks**	0.403^**^	0.213^**^	0.179^*^	0.248^**^	0.342^**^	0.425^**^
	**1-year**	0.405^**^	0.288^**^	0.364^**^	0.605^**^	0.523^**^	0.413^**^

Correlation made using Pearson’s Rank Correlation

**. Correlation is significant at the 0.01 level (2-tailed)

*. Correlation is significant at the 0.05 level (2-tailed)

## Discussion

While the EQ-VAS and Oxford Scores present different perspectives of function prior to arthroplasty, comparative analysis between how TKA and THA patients responded to these surveys in the pre-operative stage (r_s_ = 0.271, r_s_ = 0.363, respectively) suggests a only weak relationship between the two measures. The correlation between how patients answered the OKS and EQ-VAS marginally increased at 1-year (r_s_ = 0.505), and remained relatively stable between the OHS and EQ-VAS (r_s_ = 0.408). Deeper analysis of the two scales is restricted due to the limited nature of the data captured by the EQ-VAS, which is not solely limited to joint-related health by its design, however it appears as though there exists only a poor relationship between the two measures.

The FJS aims to measure “awareness” of a person’s joint by questioning their tendency to think about their joint during activities of daily living [27]. It differs from other scales as it is not site-specific, but rather may be used for any joint related pain. FJS scores post-THA achieved in this study were largely similar or slightly lower than those described in other cohorts [28] and 1-year post-TKA FJS scores were generally worse than those in the literature [29–31]. Present literature has suggested that it may be more responsive to change in patients with higher post-operative function, as it has been found to have less of a ceiling effect than other PROMs [29, 32]. In this study, there was significant difference in FJS between those who had a medium to high function (Oxford scores of 27–41, and > 41 respectively) at 1-year post operation. Cohen’s D and Eta squared demonstrated function had a large effect on FJS – supporting that it may be a better tool to identify changes in those with already good function or scoring highly in other measures. As data from the present study has demonstrated moderate FJS agreeance with the OKS and OHS across all data capture points – the collection of the two PROM scores is likely excessive in most circumstances.

OKS, OHS, EQ-VAS and the FJS PROM scores from this study demonstrate positive short-term and intermediate outcomes following TKA and THA, at our centre. There was a significant increase in scores across all three capture points: pre-operatively, six-weeks and 12-months post-operation - in each scale. Minimum clinically important difference (MCID) is a threshold for the smallest amount a PRO measure may change, yet the patient still perceives a benefit to health [33]. It is useful as a measure, as it considers clinical significance, rather than simply statistical. In this study, the average 1-year OKS increases exceeded MCID thresholds of 2.2–4.9 described in the literature [34–36]. Average change in OKS in this patient cohort was 16.86, with 83.5% (*n* = 232) patients improving by at least 5 points. Holtz, et al. identified a minimum important difference of 10.8 for FJS score in 1-year post-TKA patients [37]. The mean difference for this measure in our cohort was 36.14, and 71.3% of patients reported an improvement of at least 10.8 (*n* = 191). The number of patients meeting TKA MCIDs in this study compares well with patients self-reporting a degree of satisfaction on the 5-point Likert scale (77.2%). Average 1-year increase in OHS was 22. 59 - well above the reported MCID of 6.1-9 [36, 38]. 89.7% (*n* = 175) of patients improved by 9 points or more. Although no published research currently reports the MCID 1-year post-THA for the FJS, at 6-months the MCID has been identified as 8.1 [39]. While not a direct comparison - at 6-weeks post-THA, average FJS scores had increased by 34.5, and 82.8% (*n* = 154). Both measures compare closely to satisfaction post THA (88.5%).

The OKS/OHS is first and foremost a measure of pain and joint dysfunction as reported by patients. Higher scores, and the number of patients reaching minimal clinically important differences indicates most patients have improved pain and reduced dysfunction in their joints, at 6 -weeks and 1-year post-operation. Comparing internationally, mean post-operative OKS and OHS scores at one year are consistent (to within one standard deviation) with studies in the United Kingdom for both of our THA and THA groups [28, 40]. Knee EQ-VAS post-operative scores also reflect international studies [27] – suggesting our patient’s overall perception of health is comparable to other centres. As this measure is non-specific, improvements demonstrated relative to pre-operative levels cannot be attributed directly to joint replacement. Increases in FJS post-TKA were modest in this cohort and resulted in post-operative scores lower than those reported in literature. As the FJS is a measure of joint awareness, smaller improvements in scores within this cohort indicate that while surgery does improve overall disease morbidity, there still exist barriers preventing patients from returning to a pre-morbid level of function. Conversely, post-THA FJS averages were similar other centres.

In general, patients in our datasets had lower comorbidity scores but higher BMIs, with a cohort that was, on average, obese. Overall rates were consistent with nationally documented averages for this age-group in 2018 [41] and suggests consistency with nationally documented risk factors and standards for osteoarthritis in older adults being more common in obese individuals [42]. This consistency between our cohort of patient’s preoperative demographics and morbidity, and those of the national cohort of patients receiving the same surgical interventions increases the overall generalisability of the data presented.

In our datasets, the use of the OHS and EQ-VAS in hip arthroplasty patients highlighted that patients who were more obese may have poorer functional capacity pre-operatively. This correlation was weak, however, and high BMI preoperatively did not correlate with worse-outcomes as at 6-weeks post-operation, or 1-year post-operation as measured with OHS or FJS. It has been historically suggested that non-obese patients have more ideal recoveries in terms of pain and disability after lower limb arthroplasties and that obese patients are more prone to perioperative complications [42, 43], but data surrounding recovery following arthroplasty are mixed. A 2020 meta-analysis of 80,798 knee arthroplasty patients concluded that obesity did not account for poorer post-operative outcomes at an average follow-up of five years but comparable functional recoveries to non-obese patients [45]. For the TKA cohort, similarly weak correlations were found between pre-operative OKS and EQ-VAS PROMS, and CCI. When considering future use of PROMs in knee arthroplasty patients, it may be relevant to consider the effect CCI and other co-morbid factors may affect patients’ scorings in these scales. Satisfaction post-operatively was measured using a five-point Likert scoring system in ascending order from one to five of; (1) very dissatisfied, (2) somewhat dissatisfied, (3) neither satisfied nor dissatisfied, (4) somewhat satisfied and (5) very satisfied. Previous analysis on national-level data collected by the Australian Orthopedic Association National Joint Replacement Registry (AOANJRR) from between July 2018 and April 2020, demonstrated 86.5% of patients were satisfied post-THA, and 80.8% satisfied post-TKA [44]. Locally data collected in the present study demonstrates 88.5% of patients were either ‘very satisfied’ or ‘satisfied’ 12 months post-THA, and 77.2% satisfaction post-TKA. The number of patients who responded as being ‘dissatisfied’ or ‘very dissatisfied’ post-THA at 1-year was lower in our centre (4.0%) in comparison to a national Australian cohort (9.7%) [44]. Patients responding either as ‘dissatisfied’ or ‘very dissatisfied’ 1-year post-TKA was also lower within our department, at 8.7% relative to a national rate of 10.5%, and international figures between 16 and 20% [13, 46]. The relatively small differences in satisfaction relative to national averages suggest that our department is performing as expected.

For 2018, new standardized post-operative protocols have been introduced, including standardized physiotherapy and pain relief. These are changes that have been demonstrated in literature in the past to result in improved joint function, and thereby satisfaction [16, 47, 48]. In our dataset was a notable moderate positive linear correlation between PROM scores and satisfaction in both TKA and THA group (Table 5). This correlation was strongest in the Oxford Scores (both hip and knee). As a measure that capture patients experience of the pain, and function of their joints [7] – this is suggestive of how important pain and function are to overall satisfaction of our patients post-operatively. However, the impact of our post-operative protocols these on patient satisfaction/dissatisfaction in the present study is limited by the lack of a comparator group [47]. Relatively weak positive correlation between satisfaction and FJS score suggests that there are other factors affecting patient satisfaction beyond joint awareness alone.

In our data there is an obvious discrepancy between post-operative TKA and THA patient satisfaction. This discrepancy is widely exhibited and has been previously discussed in literature. An important factor which may contribute to this is the complex anatomy of the knee joint leading to a greater number of complications and inferior function [47]. This may lead to a failure to reach pre-operative expectations for post-operative joint function – a factor known to influence a patient’s opinion on their arthroplasty [50]. Failure to achieve expected levels of function been shown to result in increased dissatisfaction, although this applies to both hip and knee replacements [48, 51] Additionally, limitations inherent to the single-dimensional 5-point satisfaction questionnaire used limit further analysis of patient perceived factors contributing to satisfaction and dissatisfaction in this instance.

Although satisfaction scores were overall high in the six-week and one-year mark for THA (90.96%, 88.44%) and TKA (77.6%, 77.2%), this data provides restricted insight into satisfaction, and does not correlate with any of the measured PROM scales. This suggests that PROMs may provide a more wholistic and multidimensional insight into overall patient opinion. To this end other institutions have implemented self-administered satisfaction scales, comprising of four-item questionnaires exploring pain, function, and likelihood to have another operation [49].

Data on certain pre-existing psychological diagnoses – including anxiety and depression - associated with somewhat poorer outcomes and PROM scores, was not collected [52]. In further research it would be beneficial to explore any association between scales at different timepoints and when considering other such unaccounted confounders. Another limitation of this research is the fact that data was manually transcribed from paper records into electronic records systems, and that the possibility of errors in transcription could not be accounted for during analysis. In future research surrounding the use of PROMs in our institution, it may be worth initiating electronic data collection from patients and eliminating this further opportunity for error. It is already anticipated further research will compare PROM data collected by the AOANJRR and locally collected data. Furthermore, it is of note that in this dataset patient outcomes and satisfaction must be considered within the learning-curve of a teaching hospital whereby surgeries may not always be performed by consultant surgeons, but surgical trainees under supervision with varying degrees of experience. This variance of intra-operative proficiency may reduce the overall generalisability with non-teaching centres.

## Conclusion

This study provides an early insight from the use of the Oxford Knee Score, Oxford Hip Score, the EuroQol-Visual Analogue Scale and the Forgotten Joint Score as patient-reported outcome measures in primary joint arthroplasty of the hip and the knee. Pairwise comparison suggests that the arthroplasty program in our hospital is overall successful when measured by these scales, as there is significant improvement from pre-operative scores in each PROM by 6-weeks and 1-year. Improvements greater than the minimum clinically important difference was seen in the majority of patients. Patient comorbidity and body mass was poorly associated with pre-operative OKS and EQ-VAS in knees and by the FJS in hips, and did not appear to be significantly associated with post-operative outcomes. It also provides evidence of moderate positive correlation between both Oxford Scores and FJS. Weaker correlation was demonstrated between the EQ-VAS, and all other scores. Overall, 1-year post-operative TKA and THA satisfaction for our department is generally in line with that previously described in the Australian population. Moderate positive correlation between PROMs, and satisfaction were also demonstrated supporting the current understanding of the importance of post-operative pain and function to patients following orthopaedic surgery. However, the lack of a comparator prevents any conclusions being drawn regarding our own post-operative protocols.

## Electronic supplementary material

Below is the link to the electronic supplementary material.

Supplementary Material 1

Supplementary Material 2

Supplementary Material 3

## Data Availability

The raw datasets generated and/or analysed during the present study are unavailable due to identifiable patient data.

## Abbreviations

AOANJRR: Australian Orthopedic Association National Joint Replacement Registry
CCI: Charleston Comorbidity Index
EQ-VAS: EuroQol-Visual Analogue Scale
FJS: Forgotten Joint Score
FMC: Flinders Medical Centre
ICHOM: International Consortium for Health Outcome Measurement
MCID: Minimum clinically important difference
OHS: Oxford Hip Score
OKS: Oxford Knee Score
PROMs: Patient reported outcome measures
SALHN: Southern Adelaide Local Health Network
THA: Total-hip arthroplasty
TKA: Total-knee arthroplasty
