# Supplementary material for: Early insights from the routine use of patient reported outcome measures in elective hip and knee arthroplasty at a public teaching hospital in South Australia
Source: J Patient Rep Outcomes. 2024 Nov 12;8:131. doi: 10.1186/s41687-024-00807-8 (PMC11557799; doi:10.1186/s41687-024-00807-8)
Supplement: Supplementary file 1 — Supplementary Material 1 [file 41687_2024_807_MOESM1_ESM.pdf]

Southern Adelaide Local Health Network — Clinical protocol

Arthroplasty Hip & Knee - elective

Division: SAPOM | **Clinical Unit: Orthopaedic Surgery** | **MR:334**

Outpatients – elective surgery waiting list
- ☐ Consent and admission form
- ☐ Commence Prehabilitation and discharge planning with a Clinical Nurse review in the Outpatient Unit
- ☐ Book patient in for Pre surgery Waiting List Education Session
- ☐ Provide and explain Pre Surgery Waiting List Information For Hip and Knee Replacement
- ☐ Stream patients

Stream 1 (27 hour admission)
- ☐ < 75 years of age ☐
- ☐ BMI ≤ 30
- ☐ ASA 1 or 2 (confirmed at PAC)
- ☐ Home support and motivation
- ☐ Educated early mobility and expected d/c

Stream 2 (27 hour-3 day admission)
- ☐ ASA 2 or higher managed well
- ☐ BMI ≤ 40
- ☐ Home support
- ☐ Educated early mobility and expected d/c

Stream 3 (predicted sub-acute stay)
- ☐ ASA ≥ 3
- ☐ Insufficient home support
- ☐ Consider early referral to subacute services

Preadmission Clinic (10-14 days prior to surgery)
Nursing assessment

Anaesthetic consult
- ☐ Assess for anaesthetic factors which may modify stream patient entered into

Surgical RMO assessment
- ☐ Confirm patient is consented
- ☐ Chart Pre and Post op medications orders as required
  - o Preoperative – Tranexamic acid 1 g PO stat, pantoprazole 40 mg PO stat, paracetamol 1g PO stat Dexamethasone 4mg IV on induction
  - o Postoperative – Tranexamic acid 1 g PO, pantoprazole 40 mg PO daily PO (5 days), paracetamol 1 g PO qid PO, Dexamethasone 4mg IV on induction , aperients, analgesics, antiemetics
  - o Assess and document VTE risk. Document medication specific as per Arthroplasty VTE prophylaxis protocol
  - o Prophylactic antibiotics as per SALHN Antibiotic use in orthopaedic patients protocol in joint replacement
- ☐ Order patient's own medications (document any to be withheld)
- ☐ Provide patient with Medication Management Plan
- ☐ Oxford Knee Score or Harris Hip Score obtained and recorded in PROMS (Patient Reported Outcome Measure)
- ☐ Nasal swabs - MRSA/MRO for all arthroplasty patients within 2/52 of surgery AND wound swab if non-healing wound
- ☐ MSSU
- ☐ Blood tests – CBP, ELU, LFTs, Hep C, BGL, G&S 2 units, COAGS if on warfarin (note on warfarin on pathology request), hip replacement – additional white top if donating to bone bank
- ☐ Templating x-rays (Traumacad)
- ☐ ECG if over 50 years of age
- ☐ Mupirocin 2% nasal ointment if MRSA or Staph Aureus positive – 5 days prior to surgery and morning of surgery
- ☐ Provide 2 medi-sponges for shower night before and day of surgery by patient at home with patient instructions.

Physiotherapy review and education
- ☐ Post op mobility and expectations for early mobilisation
- ☐ Functional goals established for discharge – transfers, minimum walking distance, steps
- ☐ Risk Assessment and Prediction Tool (RAPT) – additional screening for subacute referral

Occupational Therapy review and education
- ☐ Assessment of discharge needs
- ☐ Equipment prescription and provision – delivered/installed prior to admission via Appliance Officer

Day prior to surgery
- ☐ Phone call from DOSA 1 business day prior to surgery – confirm fasting and admission time

Day of Surgery (admission)

- ☐ Review pre-admission assessment and medication orders, check consent
- ☐ Fast from food for 6 hours. May have water up to 2 hours prior to surgery
- ☐ Baseline and Neurovascular observations
- ☐ Check pathology results – confirm blood is available if required
- ☐ Premedications as per preadmission order. If Vancomycin/Gentamicin ordered – cannula inserted and administrated 1 hour prior to surgery
- ☐ Completion of preop checklist and skin assessment
- ☐ 2% chlorhexidine and alcohol (tinted) skin prep

↓

Perioperative care

- ☐ Antibiotics as per SALHN Antibiotic use in orthopaedic patients protocol for joint replacement
- ☐ 2% chlorhexidine and alcohol (tinted) skin prep
- ☐ Insert IDC at induction only if having spinal anaesthetic
- ☐ Spinal Anaesthetic if clinically indicated. No nerve blocks unless prior discussion with surgeons
- ☐ Infusion of local anaesthetic and adrenaline intra op administered by surgeon
- ☐ Anaesthetist to chart post op medication orders as required.
- ☐ In recovery until patient meets discharge criteria

↓

Post-op day 0 (day of surgery)

- ☐ Following assessment (WB status, obs, motor and sensory function) attempt to mobilise with physio or orthopaedic nurse. Sit up on edge of bed night of surgery if assess to be safe and has trunk control.
- ☐ If spinal anaesthetic ensure motor and sensation returning prior to mobilising
- ☐ O2 for 24 hours
- ☐ Continue Oxygen therapy to maintain SAO2 ≥ 94%
- ☐ Diet and fluid as tolerated.
- ☐ Observations: (TPR, BP, Sa02, Sedation score , Pain score, neurovascular,) hourly for 4 hrs, 2hrly for 4 hrs, 4hrly for 48 hours
- ☐ If spinal nerve block- Observations:- 1 hourly for 8hrs then 2/24 up to for 24 hours
- ☐ Document on Lower limb neurovascular chart. Record abnormalities and action taken in case-notes
    - Vascular – Colour, warmth, pulse, capillary refill of foot and toes. If possible measure and record circumference calf include date and time. Mark measuring line
    - Movement – Dorsiflex ankle and extend toes at metatarsal phalangeal joints (Peroneal N). Plantar flex ankle and toes (Tibial N)
    - Sensation – Prick web space between 1st and 2nd toe (Deep Peroneal N), Prick medial and lateral surfaces of sole foot (Tibial N), Check sensation lateral, anterior and medial sides of thigh, document any variations on diagram of leg
- ☐ Pain – pain present on passive movement or stretch of ankle and/or toes
- ☐ Antibiotics as per perioperative orders
- ☐ Antiemetics as required
- ☐ Wound – dressing to stay in place until outpatient review, if oozing for surgeon review
- ☐ Continue VTE prophylaxis including bilateral mechanical sequential compression devices please see comments
- ☐ Abduction pillow if posterior approach, no pillow for anterior approach

PRINT WARNING: – Content is continually being revised. ALWAYS refer to the electronic copy for the latest version. Users must ensure that any printed copies of this document used for an individual patients' medical record are of the latest version.

This protocol has been developed for SALHN practice setting only. It is intended to guide practice and does not replace clinical judgement. Modification will occur according to internal audit processes and literature review. First issued March 2017

Post-op (Day 1 - discharge)

- ☐ Continue analgesia as required to ensure patient can mobilise comfortably
- ☐ Daily review by Acute Pain Service
- ☐ Remove IDC 0600 hrs
- ☐ Remove IVT 0600 hrs if oral fluids tolerated
- ☐ Remove bulky dressings
- ☐ Post op bloods - Hb, ELU and coags if on warfarin
- ☐ Observations – 4hourly
- ☐ Assess aperient requirement and effectiveness
- ☐ Continue VTE prophylaxis. Remove mechanical devices once mobilising independently (with frame)
- ☐ Transfer and mobility focused therapy intervention – twice per day
- ☐ Physiotherapy clearance re mobilisation safety for discharge
- ☐ Day 1 Multi D review - identify potential barriers to discharge by Day 2 – Refer to GEM or rehabilitation, for discharge or transfer Day 3 where appropriate
- ☐ Get dressed in loose clothing Day 1
- ☐ Encourage sitting out of bed regularly for short periods
- ☐ Occupational Therapy review by Day 1 or 2 depending on progress to confirm home equipment/discharge support

↓

Discharge preparation/ Discharge Day 1 – 3

- ☐ Order discharge medications as required including, aperients, antiemetics, analgesics and VTE prophylaxis medication as per Arthroplasty VTE protocol
- ☐ Orthopaedic OPD review – arrange for 2 weeks post op at NHS Arthroplasty dressing clinic on Tuesday, then at 6 weeks Arthroplasty OPD clinic
- ☐ Discharge Information leaflet to be given to patient
- ☐ Ensure patient knows what action to take if problems arise post discharge
- ☐ Discharge summary must be completed and sent on day of discharge
- ☐ Remove cannula prior to discharge
- ☐ Physio assessment at NHS Arthroplasty wound dressing clinic - same day as orthopaedic review for mobility assessment

↓

If > Day 3 discharge

- ☐ Focused care for as early as possible discharge
- ☐ Or transfer as per predicted subacute pathway

↓

Discharge ready when

- ☐ Meets **prehabilitation** functional goals
- ☐ Vital signs - within normal limits for individual patient
- ☐ No nausea or vomiting
- ☐ Wound - nil bleeding. No signs of infection. Leave dressing intact until Arthroplasty wound review at 2/52
- ☐ Pain - pain score 0 to 3/10 - no analgesia catch up required
- ☐ Discharge drugs and letters given to patient and Nursing discharge checklist is complete
- ☐ Arthroplasty Fellow/Registrar to approve discharge. on discussion with nursing and allied team
- ☐ Sick certificate, PATS form , Centrelink forms if required

Reference

Australian Orthopaedic Arthroplasty Society Guidelines 2019

PROTOCOL:

**Title:**	Arthroplasty Hip & Knee - elective
**Approval date:**	28/01/2020
**Next review due:**	28/01/2025
**Sponsor:**	Senior Clinical Director, Professor R. Jaarsma, Orthopaedics (SALHN)
**Author:**	Nurse Practitioner and the Orthopaedic Trauma Team
**Division of Sponsor:**	Surgery & Perioperative Medicine
**Overseeing Committee:**	Orthopaedic unit meeting
**Approved at Committee:**	SALHN Policy Procedure Guideline Protocol Committee
**Risk level:**	☐ Extreme ☐ High ☐ Moderate ✓ Low
**Evaluation scheduled:**	✓ On Audit Program ☐ Not scheduled for audit
**Title and ID of Parent SA Health Policy:**	N/A
**Summary:**	Outlines best practice care for patients undergoing elective total hip and knee replacement
**Key words:**	Arthroplasty, knee, hip, replacement, THR, TKR
**Supersedes:**	Arthroplasty Hip & Knee - elective CC1.2116 Version 2.0
**Scope:**	SALHN

National Safety and Quality Health Service Standards

Clinical Governance	Partnering with Consumers	Preventing & Controlling Infections	Medication Safety	Comprehensive Care	Communicating for Safety	Blood Management	Recognising & Responding to Acute Deterioration
X	X			X	X		

Version control and change history:

Version	Date from:	Date to:	Amendment
1.0	09/03/2017	28/01/2020	Original
2.0	28/01/2020	28/01/2023	Re-worded, Reviewed and updated
2.1	21/04/2023	28/01/2025	Review date realignment

Intranet lodgement date:	April 2023
Identifier:	CC1.2116

PRINT WARNING: – Content is continually being revised. ALWAYS refer to the electronic copy for the latest version. Users must ensure that any printed copies of this document used for an official record is the latest version.
