# Supplementary material for: Early insights from the routine use of patient reported outcome measures in elective hip and knee arthroplasty at a public teaching hospital in South Australia
Source: J Patient Rep Outcomes. 2024 Nov 12;8:131. doi: 10.1186/s41687-024-00807-8 (PMC11557799; doi:10.1186/s41687-024-00807-8)
Supplement: Supplementary file 2 — Supplementary Material 2 [file 41687_2024_807_MOESM2_ESM.pdf]

Appendix 11: Orthopaedic & Spinal Surgery

This guideline does not apply to open fractures. Refer to Plastic and Reconstructive Surgery (including open fractures) guideline

Preoperative Considerations

Consider individual risk factors for every patient including the need for prophylaxis. Antibiotic choice/dose may need to be modified according to patient factors (e.g. immune suppression, presence of prostheses, allergies, renal function, obesity, malnutrition, diabetes, malignancy, infection at another site, colonisation with multi-drug resistant bacteria and available pathology).

Consider surgical wound classification (**clean, clean-contaminated, contaminated, dirty-infected)** when determining the need for, or choice of, antibiotic prophylaxis. Refer to Surgical Antimicrobial Prophylaxis Prescribing Guideline for further information.

Pre-existing infections (known or suspected) – if present, use appropriate treatment regimen instead of prophylactic regimen for procedure but ensure the treatment regimen has activity against the organism(s) most likely to cause postoperative infection. Adjust the timing of the treatment dose to achieve adequate plasma and tissue concentrations at the time of surgical incision and for the duration of the procedure - seek advice from ID or the AMS team if unsure.

For arthroplasty procedures, consider *Staphylococcus aureus* screening (for both methicillin-susceptible and methicillin-resistant strains). If the results of screening are positive, perform decolonisation. Refer to SA Health Methicillin-resistant Staphylococcus aureus (MRSA): Infection prevention and control Clinical Guideline.

Only symptomatic patients should be screened for urinary tract infection (UTI) prior to surgery and treated if appropriate. Prophylaxis antibiotics are not required for insertion or removal of urinary catheter unless a UTI is proven.

Practice Points

Timing and administration of antibiotics

Surgical antibiotic prophylaxis must be administered before surgical incision to achieve effective plasma and tissue concentrations at the time of incision. Administration of any antibiotic after skin incision reduces effectiveness.

- IV **cefazolin** can be given over 5 minutes and should be administered no more than 60 minutes before skin incision.
- IV **gentamicin** can be given over 3 to 5 minutes and should be administered within 120 minutes before surgical incision.
- IV **metronidazole** infusion can be given over 20 minutes and should be fully administered within 120 minutes of surgical incision. Maximum plasma and tissue concentrations occur at the conclusion of the infusion.
- IV **vancomycin** infusion should be given at a rate of 1g over at least 60 minutes and 1.5g over at least 90 minutes. Vancomycin should be timed to begin 15 to 120 minutes before skin incision. This ensures adequate concentration at the time of incision and allows for any potential infusion-related toxicity to be recognised before induction. The infusion can be completed after skin incision.

Dosing in patients with obesity

- **Cefazolin**: Consider increased dose of cefazolin (3g) for adult patients weighing more than 120kg.
- **Gentamicin**: For adult patients with a body mass index 30 kg/m^2 or more, use adjusted body weight (up to a maximum of 100kg) to calculate the gentamicin dose.
- **Vancomycin**: Consider increased dose of vancomycin (1.5g) for adult patients weighing more than 80kg.

High MRSA risk (defined as history of MRSA colonisation or infection OR frequent stays or a current prolonged stay in hospital with a high prevalence of MRSA OR residence in an area or aged care facility with high prevalence of MRSA OR current residence, or residence in the past 12 months, in a correctional facility):

- Add vancomycin

Repeat dosing

A single preoperative dose is sufficient for most procedures; however repeat intraoperative doses are advisable:

- for prolonged surgery (more than 4 hours from the time of first preoperative dose) when a short-acting agent is used (e.g. cefazolin dose should be repeated after 4 hours), OR
- if major blood loss occurs (e.g. more than 1500 mL in adults), following fluid resuscitation.

When measuring the time to a second intraoperative dose, measure the interval from the time of the first preoperative dose rather than the surgical incision time.

Cement

The use of antimicrobial-impregnated cement for fixation of the prosthetic device is common practice; however there is insufficient strong evidence to inform recommendations regarding the choice of antimicrobial, or concentration of antimicrobial to be in reconstituted cement. Seek ID advice.

Recommended Prophylaxis

Surgery	Recommended Prophylaxis	High Risk Penicillin / Cephalosporin Allergy*
**Primary Total Hip Replacement** (THR)<br><br>**Total Knee Replacement** (TKR)	**cefazolin 2g** IV<br><br>THEN (postoperative):<br>**cefazolin 2g** IV 8-hourly for up to 2 further doses (see NOTE 2 below)<br><br>High risk of MRSA infection :<br>**ADD vancomycin 1g** IV infusion (**1.5g** for patients more than 80kg **actual body weight**)	**vancomycin 1g** IV infusion (1.5g for patients more than 80kg **actual body weight**)<br><br>**POST-OP**: nil antibiotics required

Recommended Prophylaxis

Surgery	Recommended Prophylaxis	High risk Penicillin / Cephalosporin Allergy*
**Patients requiring revision / re-operation (joint replacement)**	**cefazolin 2g** IV<br>PLUS<br>**vancomycin 1g** IV infusion (**1.5g** for patients more than 80kg **actual body weight**)<br><br>THEN (postoperative):<br>**cefazolin 2g** IV 8-hourly for a further 2 doses<br>PLUS<br>**vancomycin 1g** IV infusion (**1.5g** for patients more than 80kg **actual body weight**) **single dose** given 12 hours after initial dose	**vancomycin 1g** IV infusion (**1.5g** for patients more than 80kg **actual body weight**)<br><br>THEN (postoperative):<br>**vancomycin 1g** IV infusion (**1.5g** for patients more than 80kg **actual body weight**) **single dose** given 12 hours after initial dose
	**Note: Pre-existing infections (known or suspected)** – if present, use appropriate treatment regimen instead of prophylactic regimen for procedure. Doses should be scheduled to allow for re-dosing just prior to skin incision.	
**Routine arthroscopic procedures not involving insertion of prosthetic material (e.g. pins, plates) or avascular tissue**	Prophylaxis NOT recommended	
**Spinal procedures**	**cefazolin 2g** IV<br><br>High risk of MRSA infection:<br>**ADD vancomycin 1g** IV infusion (**1.5g** for patients more than 80kg **actual body weight**)	**vancomycin 1g** IV infusion (**1.5g** for patients more than 80kg **actual body weight)**
**Internal fixation of fractures of large bones**<br>**Procedures involving insertion of prosthetic or allograft material**<br>**Other (closed) internal fixation**	**cefazolin 2g** IV<br><br>High risk of MRSA infection:<br>**ADD vancomycin 1g** IV infusion (**1.5g** for patients more than 80kg **actual body weight**)	**vancomycin 1g** IV infusion (**1.5g** for patients more than 80kg **actual body weight**)
**Lower limb amputation**	**cefazolin 2g** IV<br><br>If limb is ischaemic:<br>**ADD metronidazole 500mg** IV infusion<br><br>High risk of MRSA infection:<br>**ADD vancomycin 1g** IV infusion (**1.5g** for patients more than 80kg **actual body weight**)	**vancomycin 1g** IV infusion (**1.5g** for patients more than 80kg **actual body weight**)<br>PLUS<br>**gentamicin 2mg/kg** IV (for procedures likely to continue for longer than 6 hours, consider using a **5mg/kg** dose)<br><br>If limb is ischaemic:<br>**ADD metronidazole 500mg** IV infusion

***High risk penicillin/cephalosporin allergy:** History suggestive of high risk (e.g. anaphylaxis, angioedema, bronchospasm, urticaria, DRESS/SJS/TEN)

NOTE 1: BONE CEMENT

There is limited RCT evidence available to inform recommendations on the choice or dose of antibiotic in primary or secondary arthroplasty. Meta-analyses of available evidence report differing outcomes dependent upon inclusion criteria of trials. The evidence for antibiotic-impregnated bone cement in reducing deep infection rates appears stronger for secondary/revision procedures compared to primary arthroplasty. Other factors such as the use of appropriate systemic prophylaxis, laminar air-flow in the operating room, surgical proficiency and patient co-morbidities impact infection rates, and the comparative outcomes in studies investigating the efficacy of antibiotic-impregnated bone cement.

NOTE 2: POST-OPERATIVE DOSING (JOINT REPLACEMENT)

Although a single preoperative dose of surgical antibiotic prophylaxis is expected to be sufficient to prevent postoperative infection following total hip or knee arthroplasty, there is insufficient evidence to show that a single dose of prophylaxis is as effective as 24 hours of prophylaxis. Postoperative doses can be considered but **prophylaxis should not continue beyond 24 hours**.

Postoperative Care

Except where included above, postoperative antibiotics are NOT indicated unless infection is confirmed or suspected, regardless of the presence of surgical drains. If infection is suspected, consider modification of antibiotic regimen according to clinical condition and microbiological results.

Definitions / Acronyms

**AMS**	Antimicrobial Stewardship	**DRESS**	Drug rash with eosinophilia and systemic symptoms
**ID**	Infectious Diseases	**IV**	Intravenous
**MRSA**	Methicillin-resistant *Staphylococcus aureus*	**SJS / TEN**	Stevens-Johnson syndrome / Toxic epidermal necrolysis

References

Antibiotic Expert Groups (2019). Therapeutic Guidelines: Antibiotic. Version 16. Melbourne, Therapeutic Guidelines Limited.

Berbari E, Baddour L. Prevention of prosthetic joint and other types of orthopedic hardware infection (2020). In: Sexton DJ (Ed), UpToDate, Waltham, MA. [www.uptodate.com]. Accessed April 2021

Bratzler, D., E. P. Dellinger, K. M. Olsen, T. M. Perl, P. G. Auwaerter, M. K. et al. (2013) "Clinical practice guidelines for antimicrobial prophylaxis in surgery". Am J Health Syst Pharm 70:195-283.

Kleppel, D., J. Stirton, et al., Antibiotic bone cement's effect on infection rates in primary and revision total knee arthroplasties. World J Orthop, 2017. 8(12): p. 946-55

Slobogean, GP, Kennedy, SA, Davidson, D, O'Brien, PJ. (2008). "Single versus multiple-dose antibiotic prophylaxis in the surgical treatment of closed fractures: a meta-analysis". J Orthop Trauma 22:264-09

Volgt, J., Mosier, M., Darouiche, R. (2015) “Systematic Review and Meta-analysis of Randomized Controlled Trials of Antibiotics and Antiseptics for Preventing Infection in People Receiving Primary Total Hip and Knee Prostheses”. Antimicrobial Agents and Chemotherapy 59 (11):6696-6707.

Zhang, J., X.-Y. Zhang, et al., Antibiotic-impregnated bone cement for preventing infection in patients receiving primary total hip and knee arthroplasty. Medicine and Health, 2019. 98: p. 49.

Endorsed by South Australian expert Advisory Group on Antibiotic Resistance (SAAGAR). Last reviewed and amended December 2021.

SAAGAR has endeavoured to ensure that the information in this publication is accurate at the time of writing; however, it makes no representation or warranty to this effect. SAAGAR disclaims all liability for any claims, losses, damages, costs and expenses suffered or incurred as a result of reliance on this publication.
