# Supplementary material for: Early insights from the routine use of patient reported outcome measures in elective hip and knee arthroplasty at a public teaching hospital in South Australia
Source: J Patient Rep Outcomes. 2024 Nov 12;8:131. doi: 10.1186/s41687-024-00807-8 (PMC11557799; doi:10.1186/s41687-024-00807-8)
Supplement: Supplementary file 3 — Supplementary Material 3 [file 41687_2024_807_MOESM3_ESM.docx]

| **Appendix 3: Pairwise comparison of mean pre-operative PROM scores to (2) 6-weeks post-and (3) 1-year post-TKA and THA** | | | | | | |
| --- | --- | --- | --- | --- | --- | --- |
| **TKA Cohort** | | | | | | |
| **Oxford Knee Score** | | | | | | |
| (I) time | (J) time | Mean Difference (I-J) | Std. Error | Sig.^b^ | 95% Confidence Interval for Difference | |
|  |  |  |  |  | Lower Bound | Upper Bound |
| 1 (PO) | 2 (6W) | -9.78^*^ | .59 | <.001 | -11.20 | -8.36 |
|  | 3 (1Y) | -16.86^*^ | .68 | <.001 | -18.50 | -15.23 |
| **Forgotten Joint Score** | | | | | | |
| 1 (PO) | 2 (6W) | -18.22^*^ | 1.61 | <.001 | -22.10 | -14.33 |
|  | 3 (1Y) | -36.14^*^ | 1.96 | <.001 | -40.85 | -31.42 |
| **EQ-VAS** | | | | | | |
| 1 (PO) | 2 (6W) | -5.01^*^ | 1.50 | .003 | -8.63 | -1.38 |
|  | 3 (1Y) | -10.64^*^ | 1.37 | <.001 | -13.94 | -7.33 |
| **THA Cohort** | | | | | | |
| **Oxford Hip Score** | | | | | | |
| (I) time | (J) time | Mean Difference (I-J) | Std. Error | Sig.^b^ | 95% Confidence Interval for Difference | |
|  |  |  |  |  | Lower Bound | Upper Bound |
| 1 (PO) | 2 (6W) | -16.13^*^ | .83 | <.001 | -18.12 | -14.13 |
|  | 3 (1Y) | -22.59^*^ | .77 | <.001 | -24.46 | -20.73 |
| **Pairwise Comparison – Forgotten Joint Score** | | | | | | |
| 1 (PO) | 2 (6W) | -34.50^*^ | 2.38 | <.001 | -40.25 | -28.75 |
|  | 3 (1Y) | -49.87^*^ | 2.36 | <.001 | -55.59 | -44.16 |
| **Pairwise Comparison – EQ-VAS** | | | | | | |
| 1 (PO) | 2 (6W) | -10.93^*^ | 1.32 | <.001 | -14.14 | -7.73 |
|  | 3 (1Y) | -13.51^*^ | 1.41 | <.001 | -16.94 | -10.08 |
| Based on marginal means.  *. The mean difference is significant at the .05 level.  Adjustment for multiple comparisons: Bonferroni. | | | | | | |

| **Appendix 4: Correlations between PROM scores, and Weight, BMI and CCI covariates** | | | | | | | | | |
| --- | --- | --- | --- | --- | --- | --- | --- | --- | --- |
| **THA Cohort** | | | | | **TKA Cohort** | | | | |
|  |  | **Age** | **BMI** | **CCI** |  |  | **Weight** | **BMI** | **CCI** |
| OHS Pre | Correlation Coefficient | -.128 | **-.162^*^** | .131 | OKS Pre | Correlation Coefficient | .032 | -.034 | **.169^**^** |
|  | Sig. (2-tailed) | .070 | .026 | .064 |  | Sig. (2-tailed) | .603 | .587 | .005 |
| OHS 6W | Correlation Coefficient | .034 | -.132 | .056 | OKS 6W | Correlation Coefficient | -.110 | -.092 | .029 |
|  | Sig. (2-tailed) | .630 | .073 | .431 |  | Sig. (2-tailed) | .074 | .136 | .626 |
| OHS 1Y | Correlation Coefficient | -.085 | -.060 | .093 | OKS 1Y | Correlation Coefficient | **-.150^*^** | -.103 | .057 |
|  | Sig. (2-tailed) | .232 | .416 | .194 |  | Sig. (2-tailed) | .015 | .097 | .341 |
| FJS Pre | Correlation Coefficient | **.166^*^** | **-.150^*^** | -.138 | FJS PRE | Correlation Coefficient | -.031 | -.086 | -.003 |
|  | Sig. (2-tailed) | .020 | .044 | .054 |  | Sig. (2-tailed) | .610 | .165 | .954 |
| FJS 6W | Correlation Coefficient | .125 | -.134 | -.073 | FJS 6W | Correlation Coefficient | -.061 | -.056 | .027 |
|  | Sig. (2-tailed) | .084 | .074 | .314 |  | Sig. (2-tailed) | .333 | .379 | .661 |
| FJS 1Y | Correlation Coefficient | -.012 | -.116 | .021 | FJS 1Y | Correlation Coefficient | -.050 | -.031 | -.050 |
|  | Sig. (2-tailed) | .875 | .127 | .774 |  | Sig. (2-tailed) | .424 | .625 | .416 |
| EQVAS Pre | Correlation Coefficient | .064 | **-.291^**^** | .067 | EQVAS PRE | Correlation Coefficient | -.043 | **-.155^*^** | **.125^*^** |
|  | Sig. (2-tailed) | .388 | <.001 | .364 |  | Sig. (2-tailed) | .504 | .016 | .046 |
| EQVAS 6W | Correlation Coefficient | .064 | **-.149^*^** | .055 | EQVAS 6W | Correlation Coefficient | .075 | .004 | .059 |
|  | Sig. (2-tailed) | .374 | .047 | .452 |  | Sig. (2-tailed) | .229 | .955 | .337 |
| EQVAS 1Y | Correlation Coefficient | -.037 | -.116 | .069 | EQVAS 1Y | Correlation Coefficient | -.060 | -.090 | .102 |
|  | Sig. (2-tailed) | .610 | .122 | .341 |  | Sig. (2-tailed) | .349 | .156 | .097 |

| **Appendix 5: Satisfaction at 6-weeks and 1-year post-TKA and THA (Descriptive Statistics)** | | | | | | | |
| --- | --- | --- | --- | --- | --- | --- | --- |
|  | **Interval** | **Counts** | | | | | |
|  |  | Missing | Very Unsatisfied, N (%) | Unsatisfied, N (%) | Neither Satisfied or Unsatisfied, N (%) | Satisfied, N (%) | Very Satisfied N (%) |
| **TKA** | 6-weeks | 18 | 1 (0.4) | 16 (6.0) | 43 (16.1) | 107 (40.1) | 100 (37.5) |
|  | 1-year | 22 | 4 (1.5) | 19 (7.2) | 37 (14.1) | 78 (29.7) | 125 (47.5) |
|  |  | **Descriptive Statistics** | | | | | |
|  |  | N | Mean | Standard Deviation | Skewedness | Std Error of Skewedness | Kurtosis Statistic |
|  | 6-weeks | 267 | 4.082 | .896 | -.792 | .149 | .056 |
|  | 1-year | 263 | 4.144 | 1.010 | -1.072 | .150 | .399 |
| **THA** |  | **Counts** | | | | | |
|  |  | Missing N (%) | Very Unsatisfied, N (%) | Unsatisfied, N (%) | Neither Satisfied or Unsatisfied, N (%) | Satisfied, N (%) | Very Satisfied N (%) |
|  | 6-weeks | 20 | 1 (0.5) | 0 (0) | 16 (8.6) | 47 (25.4) | 121 (65.4) |
|  | 1-year | 30 | 3 (1.7) | 4 (2.3) | 13 (7.4) | 30 (17.1) | 125 (71.4) |
|  |  | **Descriptive Statistics** | | | | | |
|  |  | N | Mean | Standard Deviation | Skewedness | Std Error of Skewedness | Kurtosis Statistic |
|  | 6-weeks | 185 | 4.55 | 0.698 | -1.636 | .179 | 3.108 |
|  | 1-year | 175 | 4.54 | 0.862 | -2.173 | .184 | 4.706 |
